# Initial damage produced by a single 15-Gy x-ray irradiation to the rat calvaria skin

**DOI:** 10.1186/s41747-020-00155-4

**Published:** 2020-06-05

**Authors:** Matheus da Silva Santin, José Koehler, Danilo Massuia Rocha, Camila Audrey dos Reis, Nadia Fayez Omar, Yasmin Fidler, Maria Albertina de Miranda Soares, José Rosa Gomes

**Affiliations:** 1 Universidade Estadual de Ponta Grossa, DEBIOGEM, Carlos Cavalcanti, Campus Uvaranas, Ponta Grossa, Paraná, 84040060 Brazil; 2 Southern Paraná Oncology Institute (ISPON), Cel. Francisco Ribas, 638 - Ponta Grossa, Paraná, Brazil

**Keywords:** Collagen, Radiobiology, Rats, Skin, X-rays

## Abstract

**Background:**

Calvaria skin has a reduced thickness, and its initial damage produced by irradiation was scarcely reported. We aimed to identify the initial effects of x-ray irradiation in the rat calvaria skin.

**Methods:**

After approval by the Animal Ethical Committee, calvaria skin sections of five Wistar rats per time point were evaluated on days 4, 9, 14, and 25 following a single 15-Gy x-ray irradiation of the head. The control group was composed of five rats and evaluated on day 4. Sections were assessed using hematoxylin-eosin and Masson’s trichrome staining for morphology, inflammation, and fibrosis. Fibrosis was also evaluated by the collagen maturation index from Picrosirius red staining and by cell proliferation using the immunohistochemistry, after 5-bromo-2-deoxyuridine intraperitoneal injection.

**Results:**

In irradiated rats, we observed a reduction in epithelial cell proliferation (*p* = 0.004) and in matrix metalloproteinase-9 expression (*p* < 0.001), an increase in the maturation index, and with a predominance in the type I collagen fibers, on days 9 and 14 (1.19 and 1.17, respectively). A progressive disorganization in the morphology of the collagen fibers at all time points and changes in morphology of the sebaceous gland cells and hair follicle were present until day 14.

**Conclusions:**

The initial damage produced by a single 15-Gy x-ray irradiation to the rat calvaria skin was a change in the normal morphology of collagen fibers to an amorphous aspect, a temporary absence of the sebaceous gland and hair follicles, and without a visible inflammatory process, cell proliferation, or fibrosis process in the dermis.

## Key points

- A single 15-Gy x-ray irradiation to rat calvaria skin induced temporary atrophy in the sebaceous glands and hair follicles.
- A single 15-Gy x-ray irradiation to rat calvaria skin changed the organized morphology of collagen fibers.
- A single 15-Gy x-ray irradiation to rat calvaria skin did not induce inflammation or fibrosis.

## Background

Different treatments have been used to reduce the metastasis process, such as chemotherapy, surgery, and radiotherapy, which are used worldwide. Radiotherapy induces cancer cell death by a chemical action mainly due to the generation of the free radicals, which are produced by the breaking of the water molecules present in the extracellular matrix of the cancer tissue [1]. The damage produced by radiotherapy in the healthy and cancer cells as well as in the tissues near to or away from the cancer region was called side effects [2] and is still a problem to be solved in oncology. In this context, since the skin is the first organ hit by irradiation, it also presents several reactions denominated acute and late side effects [3]. The acute effects such as erythema occur immediately or within a few days after irradiation [4] and are dose dependent. In 95% of the cases, patients present some kind of acute reaction, such as hypersensitiveness, itchiness, pain, and some level of infection [5, 6].

On the other hand, the late side effects may occur in human skin around 12 or 16 weeks or even years after the end of radiotherapy, but in the experimental animal model, it starts around 4 weeks [7, 8]. The late side effects produced by radiation in the skin that covers most of the body are described as chronic inflammation and afterwards fibrosis [9, 10], which is an increase in the deposition of collagen fibers due to an increase of the myofibroblast differentiation, with synthesis and deposition of collagen fibers.

Fibrosis is strongly induced by transforming growth factor-β (TGF-β) expression [11, 12], and there is no description for an efficient mechanism of downregulation for the TGF-β expression and consequently for fibrosis. Skin fibrosis is widely and well described in several experimental approaches using different radiation doses and protocols (single or fractional) in different body skin regions such as the ear, dorsal skin, and leg skin, as well as in biopsies of the human skin [13, 14]; however, for the calvaria skin, these late side effects were not still reported.

Therefore, in the present study, we aimed to identify the effects of a single 15-Gy x-ray dose to the skin calvaria of rats concerning inflammation, collagen deposition, and cell proliferation in the epidermis and dermis before the time described in the literature, defined as the beginning of fibrosis.

## Methods

The University Committee for Ethics in Animal Research approved all experiments in the study with the protocol number 3822/2011.

### Experimental design

The sample size calculation was based on a pilot experiment performed in our laboratory using three animals submitted to the same x-ray dose used in the present manuscript. In this experiment, the parameter used was the count of the number of goblet cells in the small intestine, from all groups (distributed at the same time points). The results were transformed into the square root and submitted to analysis of variance test to obtain the mean, the minimum difference between the means, and the standard deviation of the mean. These values fed the tool to determine the *n* sample in the Biestat 5.0 software of the public domain obtained from Mamirauá Institute in Brazil (https://www.mamiraua.org.br/downloads/programas/) adopting a significance level of *α* = 0.05 and a test power of 80%. The result was a minimum number of four animals per repetition. However, we decided to use five animals for each repetition, considering that some of them could die during the experimental procedures.

### Animal groups

Twenty-five adult male 3-month-old Wistar rats with a mean weight of 300 g were obtained from the UEPG animal house and kept under conventional conditions with a 12-h light/dark cycle (lights on at 06:30 am; lights off at 6:30 pm) at a room temperature between 23 and 25 °C, and received food (nutrition-balanced ration from Nuvital, Brazil) and water ad libitum. The 25 rats were distributed into 5 groups, each of them composed of 5 animals: 4 experimental groups and a control group. The experimental groups were sacrificed on days 4, 9, 14, or 25 after irradiation, the control group on day 4 after irradiation.

### Experimental procedures

**Fig. 1:**
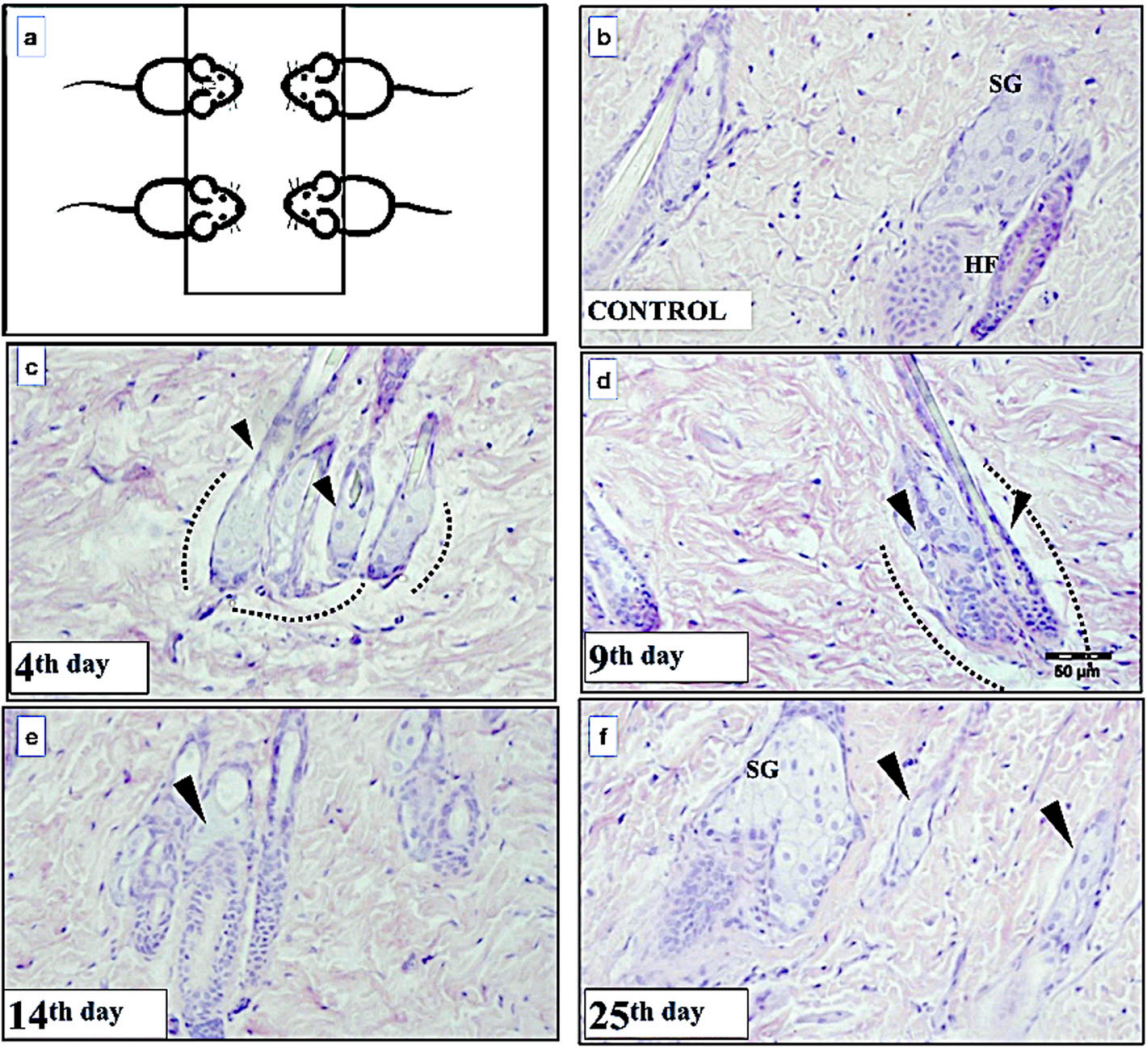
Schematic representation of the rat disposition for radiation application (**a**) and representative images of the skin morphology from hematoxylin-eosin staining after the application of x-ray irradiation. Control (**b**), day 4 (**c**), day 9 (**d**), day 14 (**e**), and day 25 (**f**). Altered sebaceous gland (arrowheads) and dotted lines show the spaces between sebaceous gland plus hair follicle from the extracellular components. *HF* Hair follicle, *SG* Sebaceous gland

A single 15-Gy x-ray dose was applied on the head of all rats in the experimental groups, placed in a ventral decubitus position (Fig. 1), using a focal distance of 100 cm and a collimation field of 40 × 40 cm with a linear accelerator 600C/D-6MV (Varian, Palo Alto, CA, USA) from the Southern Paraná Oncology Institute (ISPON), located in Ponta Grossa, State of Parana, Brazil. Before irradiation and in the day of sacrifice, all rats were injected with ketamine hydrochloride (Dopalen® Agribrands do Brasil Ltda., Paulínea, São Paulo, Brazil) at a dose of 1.0 mL/kg of body weight and xylazine hydrochloride (Rompum® Bayer S.A., São Paulo, Brazil) at a dose of 1.5 mL/kg of body weight.

For the aim of cell proliferation analyses, all the rats received an injection of 5-bromo-2-deoxyuridine (BrdU) (Sigma-Aldrich Chemical, São Paulo, Brazil) at a dose of 0.5 mg per 100 g of body weight 1 h before sacrifice. The BrdU is an analog molecule that is incorporated by cells during the S phase of the cell cycle (when deoxyribonucleic acid is being duplicated). Thus, cells in proliferative status may be detected on histological sections by immunohistochemistry to estimate the cell proliferation index (see below).

#### General procedures

**Fig. 2:**
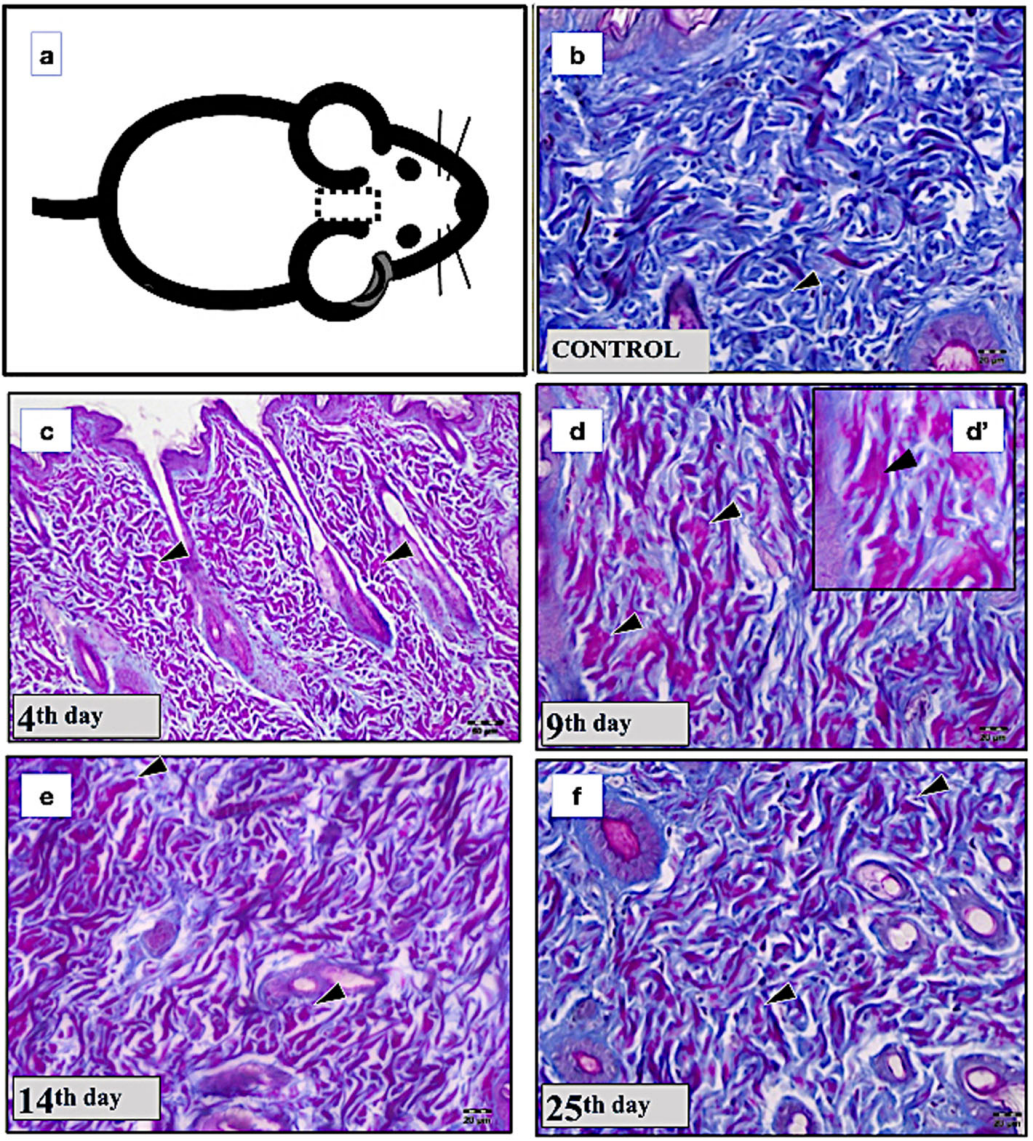
Representative images of the dermis layer of skin obtained from Masson trichrome staining for each time point assessed after x-ray irradiation. Scheme of the calvaria region in which the skin was evaluated (**a**). Control (**b**), day 4 (**c**), day 9 (**d**), detail from **d** (**d’**), day 14 (**e**), and day 25 (**f**). Note the amorphous morphology of the collagen fibers produced by the x-ray irradiation in **d’** (arrowheads)

Figure 2a indicates the region of the head from where the skin fragments were collected. Fragments were then immersed in a 2% formaldehyde 0.1 mol/L phosphate buffer at pH 7.4 for 48 h. Samples were dehydrated in alcohol and embedded in paraffin to obtain three 5-μm semiseriated sections that were mounted on slides.

#### Cell proliferation assay

Skin sections were dewaxed, rehydrated, immersed in 1 NHCl at 40 °C for 1 h, and water washed. Afterward, sections were quenched three times with a 2% hydrogen peroxide solution for 10 min each to inhibit endogenous peroxidase activity, washed in 1× phosphate buffered saline (PBS) at pH 7.4, and incubated overnight at 4 °C with monoclonal anti-BrdU antibody (2 mg/mL) (Abcam, Cambridge, UK) at a 1:500 ratio in 1× PBS at pH 7.4, containing 1% bovine serum albumin. After this time, they were washed in 1× PBS and incubated with the secondary antibody using the universal DAKO LSAB kit (Denmark A/C, Demark) for 30 min at 37 °C. After washed in 1× PBS, the sections were incubated with the diaminobenzidine tetrahydrochloride reagent (Sigma Chemical, St. Louis, USA) prepared in 1× PBS in the presence of both hydrogen peroxide and dimethyl sulfoxide. Negative control staining was performed by substituting the primary antiserum with nonimmune serum, and all sections were counterstained with hematoxylin. From each section, the proliferative index was calculated by counting the number of BrdU-positive cells present in five regions of the epidermis. The proliferative index was expressed as the mean of the proliferative cells by section.

#### Collagen morphology and inflammation assay

The morphological analyses were determined by Masson’s and hematoxylin-eosin staining to evaluate the morphology of dermal components as collagen fibers, hair follicle, and sebaceous gland and for the presence of inflammatory cells. Because authors reported that irradiation induces inflammation before the fibrosis [9, 10], we also evaluated the expression of matrix metalloproteinase 9 (MMP-9) which is an enzyme related to the final degradation of extracellular components as collagen fibers. MMP-9 is higher expressed by inflammatory cells like macrophages, neutrophils, and mast cells. For this assay, cross-sections were submitted to initial and final immunohistochemistry procedures already described for the cell proliferation assay. However, before the incubation overnight at 4 °C with monoclonal MMP-9 antibody (2 mg/mL) (Abcam, Cambridge, UK) at a 1:200 ratio, sections were immersed in 1 mM ethylenediaminetetraacetic acid at pH 8.0 at 37 °C for 8 h to induce antigen recovering. For analysis, the number of positive cells expressing MMP-9 was counted in five areas of each section by rat and time point evaluated.

#### Estimate of radiation-induced fibrosis

Skin sections were submitted to Masson’s trichrome staining to evaluate visually the morphology of collagen fibers. Fibrosis was estimated from the maturation index in sections submitted to Picrosirius red staining [15] under polarized light microscopy from which the images were taken in the magnification of × 40. The limited value established for the maturation index is 1.0, and from this value, it is possible to estimate the predominance of type I collagen fibers when the value was superior to 1.0 or of type III collagen fibers when this value was inferior to 1.0 [16, 17]. The Picrosirius staining produces an orange/red color which corresponds to type I collagen fibers, while the green color corresponds to the type III collagen fibers. After the images of the sections had been taken, they were processed to binary images using the ImageJ software (Image/ImageJ, National Institutes of Health, MD, USA), and mean areas of pixel intensity were obtained separately for orange and green colors. Then, the ratio between the pixel mean obtained from the orange colors (type I collagen) and the green color (type III collagen) was calculated. These results were called R1 for the control group and R2, R3, R4, and R5 for each time point evaluated in the experimental groups. Finally, to obtain the exact maturation index for each time evaluated, the value 1.0 was considered as constant for the control group, and from that, the maturation index was calculated using the formula

$$ \mathrm{Maturation}\ \mathrm{index}=\mathrm{R}2\times 1.0/\mathrm{R}1 $$

and thus for the other time points, respectively.

### Statistical analysis

The number of proliferative cells and MMP-9-positive cells obtained were submitted to one-way analyses of variance, using the Prism software 3.0 (GraphPad Software, Inc., La Jolla, CA, USA), with the Tukey post-test at *p* < 0.05.

## Results

The administered x-ray irradiation induced an initial atrophy of the sebaceous glands and hair follicles, a change in the normal morphology of collagen fibers to amorphous aspect, but not a visible inflammatory or fibrosis process.

Figure 1 shows representative images of the skin morphology obtained with hematoxylin-eosin staining for each time point after x-ray irradiation. Compared to the control group (Fig. 1b), the irradiation side effects may be observed in the general morphology of sebaceous glands and hair follicles on day 4 (Fig. 1c) and day 9 (Fig. 1d). The morphology of the gland cells was altered as indicated by arrows in Fig. 1c, as well as the hair follicle, which seemed reduced. A relationship loss between the sebaceous gland and the surrounding extracellular matrix (dotted lines), as well as changes in the morphology of the hair follicle, could be also observed as indicated in Fig. 1c and d. Fourteen days after irradiation, the sebaceous gland was reduced to a few cells, as indicated by the arrows in Fig. 1e, and the hair follicle was completely atrophied. On day 25 (Fig. 1f), the presence of the sebaceous gland in which the cells presented a morphology close to that of the control group was observed. Concerning the presence of inflammatory cells, the results did not demonstrate any significant influx of inflammatory cells at any time point evaluated.

In addition, the x-ray irradiation modified the morphology of collagen fibers to an amorphous aspect with changes in the collagen maturation index. Fig. 2b shows the control group with normal collagen fibers stained in blue with Masson staining. In the experimental groups, the collagen fibers had their morphology altered, presenting a red color with an amorphous aspect on day 4 and day 9 (Fig. 2c, d, and d). On day 14 and day 25, the amorphous aspects of the collagen fibers were still present (Fig. 2e, f), but some of the dermis regions showed an organization similar to that observed in the control group. Normal collagen fibers in blue color were also observed on day 25.

**Fig. 3:**
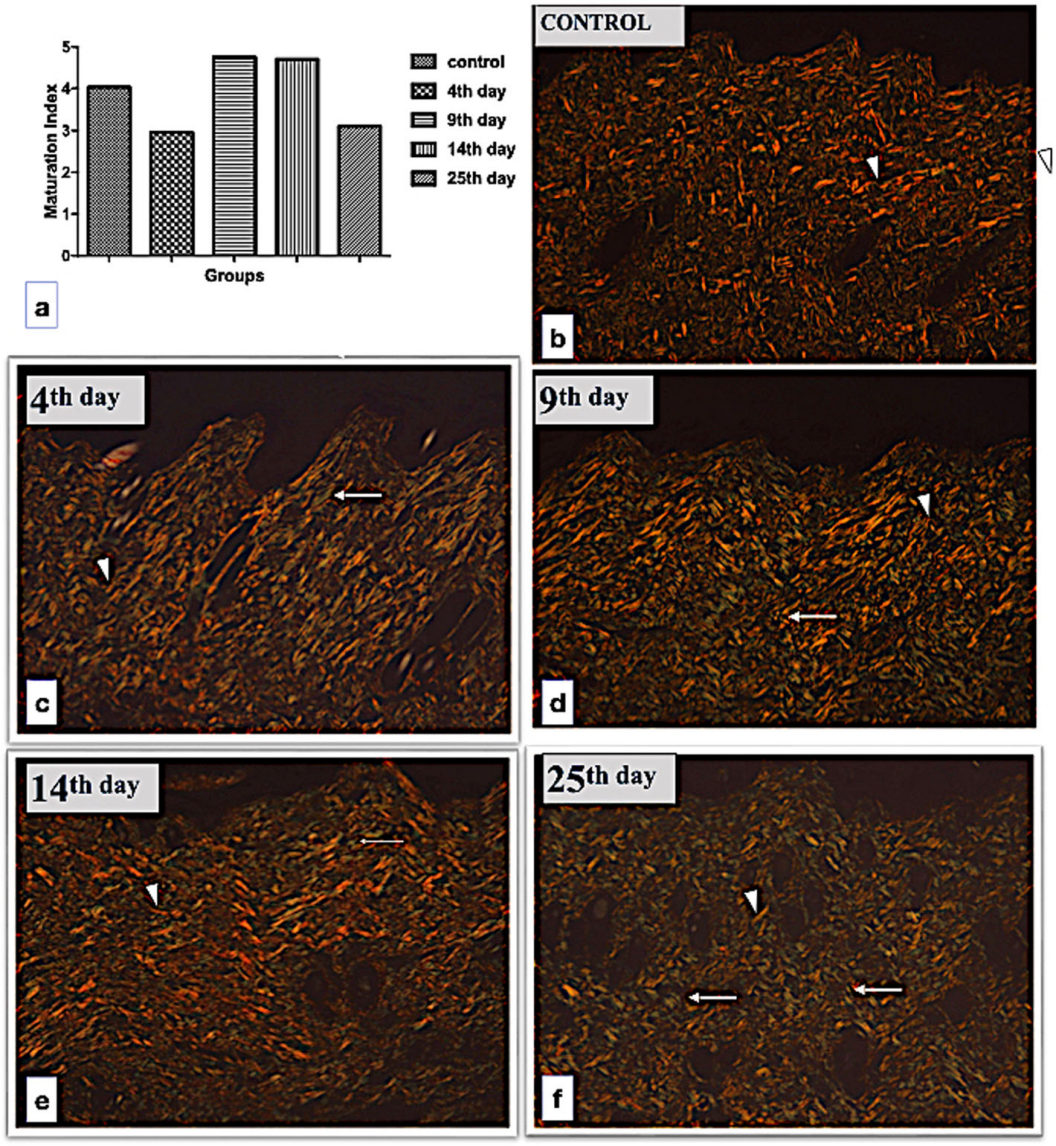
Maturation index of collagen fibers (**a**) and representative images of the dermis layer of the skin obtained from Picrosirius red staining under polarized light for each time point evaluated after x-ray irradiation. Control (**b**), day 4 (**c**), day 9 (**d**), day 14 (**e**), and day 25 (**f**). Type I collagen fibers in red (arrowhead) and type III collagen fibers in green (arrows)

Figure 3a shows the results for the maturation index, calculated from the analysis of sections stained by Picrosirius red staining, in which the value 1.0 for the control group was considered. When the pixel for type I collagen fibers was compared among the groups, we observed a significant increase on day 14 compared to day 4 (*p* = 0.034) and day 25 (*p* = 0.016). However, the maturation index showed a predominance of type I collagen fibers only on day 9 and on day 14, with values of 1.19 and 1.17, respectively. Conversely, the predominance of the type III collagen fibers occurred on day 9 and day 25, with values of 0.72 and 0.77, respectively. Figure 3b to f show representative images of the Picrosirius red staining for the time points evaluated, representing the collagen type I fibers in red (arrows) and the collagen type III in green (arrows).

X-ray irradiation reduced the epidermis cell proliferation and the MMP-9 expression in the dermis.

**Fig. 4:**
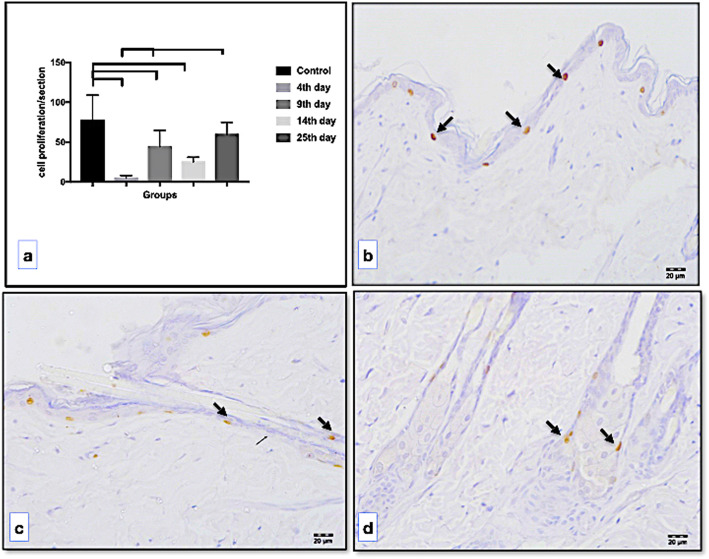
Number of proliferation cells per section in the epidermis (**a**) and representative images for 5-bromo-2-deoxyuridine-positive nucleus (arrows) in the epidermis (**b**), in the hair follicle (**c**), and at the base of a sebaceous gland (**d**)

**Fig. 5:**
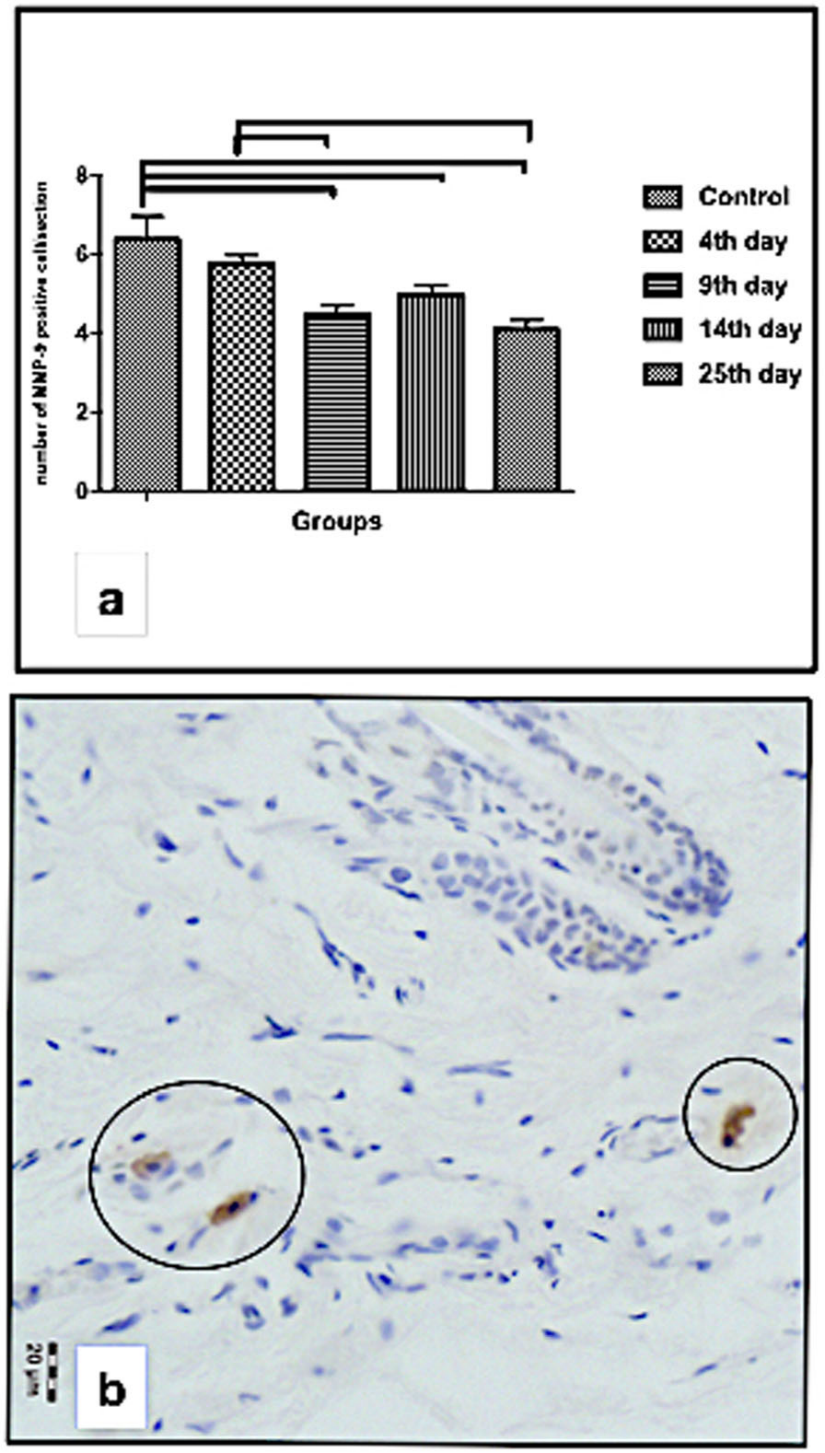
Number of metalloproteinase 9 (MMP-9)-positive cells per section in the dermis (**a**) and a representative image showing the immunostaining for (MMP-9) in the cytoplasm of the cells of the dermis of the control group (circles in **b**)

The cell proliferation index obtained in the epidermis (expressed as the number of proliferative cells per section) is presented in Fig. 4a, while representative BrdU-positive cells in the epidermis basal layer and hair follicle as well as in the sebaceous gland are shown in Fig. 4b, c, and d, respectively. Figure 5a shows that the number of MMP-9-expressing cells decrease during the time, being significantly reduced on days 4 (*p* < 0.001), 9 (*p* = 0.002), and 14 (*p* < 0.001) when compared to the control group. On days 9 (*p* = 0.003) and 25 (*p* < 0.001), the number of MMP-9-expressing cells was also significantly reduced when compared to day 4 of the experimental group, it increased significantly on day 9 (*p* = 0.003) and day 25 (*p* < 0.001) but was not significantly different on day 14 (*p* = 0.473). On days 9 and 14, the epidermis cell proliferation was not significantly different when compared to days 14 and 25 (*p* = 0.529, *p* = 0.534) respectively, as well as to day 14 compared to day 25 (*p* = 0.075). However, no BrdU-positive cells were detected at any of the time points evaluated, indicating no renewal of the dermis cells in this time period.

The expression of MMP-9 was detected in the dermis cells in the control and irradiation groups. The highest number of MMP-9-expressing cells was observed in the area close to the subcutaneous tissue. Figure 5a shows that the number of MMP-9-expressing cells decrease during the time, being significantly reduced on days 9 (*p* < 0.001), 14 (*p* = 0.002), and 25 (*p* < 0.001) when compared to the control group. On days 9 (*p* = 0.003) and 25 (*p* < 0.001), the number of MMP-9-expressing cells was also significantly reduced when compared to day 4. Figure 5b shows the representative MMP-9 immunohistochemistry in the dermis on day 9. The morphology of MMP-9-expressing cells and of their nuclei indicates that it may be a mast cell.

## Discussion

It is known that in humans after the radiotherapy ends, several late effects appear in the tissues of organs irradiated which in turn may change their functionality. These late effects, well described for the skin 16 weeks after irradiation in humans and after 4 weeks after irradiation in animal models, consist of an initial inflammatory process followed by an intense production of collagen fibers characterizing the fibrosis process [18, 19]. However, until this manuscript has been produced, similar effects in the calvaria skin were not described.

Therefore, in the present study, the calvaria skin was studied after a 15-Gy x-ray irradiation according to previous results [20], which reported this dose as appropriate for obtaining a chronic effect of radiation, and after this dose has been confirmed by the nuclear physicist from the Instituto Sul Paranaense de Oncologia (ISPON) placed in Ponta Grossa City, Paraná, Brazil.

In general, for organs and for the skin of other body parts than calvaria, fibrosis is reported after inflammation. In turn, inflammation is described as a consequence of the action of ionizing radiation which changes the macromolecules building organs and tissues, especially those from the cell membranes which are broken to generate molecules known as reactive oxygen species (ROS). ROS produce high oxidative stress that is responsible for the sequential damage of cells of the tissues and organs. For example, ROS break the endothelium of the small blood vessels to start the inflammatory cell process. The increase in oxidative stress also induces an increase in the enzymes that have the function to kidnap the ROS reducing the damages [21, 22]. Although the detection of ROS and ROS-associated enzymes can be performed by specific methods, the presence of inflammatory cells and the changes in morphology of the connective tissues (*i.e.*, of collagen fibers or vessels) already indicates that an oxidative stress is happening. Thus, after an initial response to radiation damages, the connective cells start the regeneration of the injured structures. The more visible and detectable aspect of this regeneration process is the production of new collagen fibers, *i.e.*, a fibrosis. However, after irradiation, this process occurs without appropriate control, which means that it may affect the functionality of different organs of the body [22].

On the other hand, it is known that MPPs are responsible for remodeling extracellular matrix components and are regulated by endogenous tissue inhibitors of metalloproteinases (TIMPs) [23]. The relationship between the MMPs, TIMPs, and TGF-β1 has been explored in the heart, lung, and enteritis fibrosis [24–26]. However, for the skin, this relationship has been poorly explored [27]. Thus, we evaluated the expression of MMP-9 in the calvaria skin day 4 to day 25 after the irradiation. The results showed that the number of MMP-9-positive cells in the dermis decreases from day 4 but significantly on days 9, 14, and 25 compared to controls. Although without a visible inflammatory cell influx in the dermis of the calvaria skin during this period, the MMP-9 results indicate a reduction in the remodeling of the extracellular matrix components of the dermis which may confirm the absence of a fibrosis process during these times evaluated.

In spite of this, results corroborate what has been described for the skin that covers the most regions of the body [28, 29], related with drastic but temporary atrophy of sebaceous glands and hair follicles. We found that between day 4 and day 14, these epidermal components disappeared. The reduced number of BrdU-positive cells in the basal layer indicates a precocious effect of radiation in the cell proliferative process. However, on day 25, the presence of BrdU-positive cells in the epidermis, close to sebaceous glands as well as in the hair follicle, indicates that the proliferative process was reestablished. Of note, the use of stem cells obtained from different tissues has been investigated *in vitro* and *in vivo* to induce the regenerative damage produced by radiotherapy [13, 30, 31]. Therefore, the reestablishment of the cell proliferation observed in our results suggests the presence of the stem cells in these epidermal structures or its migration from another region to assume this proliferative status. However, the dynamics of stem cells in skin submitted to radiation needs to be better investigated in our model study: the calvaria skin.

Regardless, the literature shows that a myofibroblast differentiation occurs and this causes the fibrosis process in the dermis of calvaria skin, and we did not observe cell proliferation after irradiation, playing in favor of an interruption of this process. Although it had been reported in previous studies [11, 20] that in the skin of other parts of the body, the fibrosis may occurs at least from week 4 after a single dose of 15 Gy and that it is preceded by an inflammation, we did not observe this process in the calvaria skin until the day 25. This finding was also confirmed by the reduction of the number of MMP-9-expressing cells in the dermis, MMP-9 being considered an active molecule involved in the inflammatory process. This may be partly explained because in the calvaria skin, and some other regions of the head, the low density and thickness of the subcutaneous layers containing blood vessels may reduce the generation of inflammation. However, we do not exclude the possibility of a subsequent fibrosis process starting after the time period here considered, opening the way for future studies prolonging the observation after day 25.

Concerning the extracellular matrix in the dermis, it is known that in a normal condition, the ratio of types I and III collagen fibers in covered skin remained constant throughout childhood and young adult life [32]. However, it is interesting that collagen fibrils in the dermis are hybrid molecules formed by type I and type III collagen, in which type III collagen is located at the periphery of the collagen fibrils [33, 34]. In this context, the general descriptions for the radiation effects in the dermis of skin covering most body regions are an increase in its thickness as a result of the collagen fiber deposition, an increase in the fiber diameter, and the replacement of the subcutaneous fat and skeletal muscle by a fibrotic tissue [35].

In our investigation, the Masson’s staining allowed us to detect the two types of collagen fibers in the control group (type I collagen fibers in blue; type III collagen fibers in red [35, 36]). However, in the irradiated calvaria skin, it was possible to determine a significant change in the pattern of the color of collagen fibers by Masson staining not previously well explored. Irradiation produced alterations of the collagen morphology with a deep change, going from a fibrous and waive arrangement to a complete fiber degradation in some regions, with amorphous aspect. These changes were detected at all time points, becoming more evident at day 9, suggesting that the amorphous aspect of the collagen fibers resulted from the dissociation of type III collagen, which increased the collagen maturation index for type III collagen, without any increase in the collagen synthesis. Type III collagen fibers were predominant on day 4 and day 25, and type I collagen fibers on day 9 and day 14. The predominance of both collagen fibers may be the result of their disorganization produced by the radiation and not caused by new collagen synthesis, considering that proliferation of dermal cells was never detected.

In conclusion, our findings showed that a 15-Gy x-ray irradiation to the calvaria skin evaluated until day 25 (1) changed the morphology of collagen fibers in the dermis to an amorphous aspect in few days after irradiation, (2) produced a temporary morphological damage of the sebaceous gland and hair follicles, and (3) did not determine a visible inflammatory process, cell proliferation, or fibrosis process in the dermis, differing from the results already described in the literature for the skin of other body parts.

## Data Availability

The datasets used and/or analyzed during the current study are available from the corresponding author on reasonable request.

## Abbreviations

BrdU: 5-Bromo-2-deoxyuridine
MMP-9: Matrix metalloproteinase 9
PBS: Phosphate-buffered saline
ROS: Reactive oxygen species
TGF-β: Transforming growth factor-β
